# Topological indices based VIKOR assisted multi-criteria decision technique for lung disorders

**DOI:** 10.3389/fchem.2024.1407911

**Published:** 2024-09-24

**Authors:** Tahreem Ashraf, Nazeran Idrees

**Affiliations:** Department of Mathematics, Government College University, Faisalabad, Pakistan

**Keywords:** topological indices, QSPR analysis, MCDM, lung disorders drugs, VIKOR

## Abstract

Lung disorders involve swelling, inflammation, and muscle tightening around the airways, with symptoms such as coughing, wheezing, shortness of breath, and abnormal fluid build-up. The global prevalence of these conditions is rising, highlighting the need for extensive research to alleviate their severity and symptoms. Due to the chronic nature and recurrence of these disorders, the human body often develops immunity and side effects to certain medications. Therefore, developing novel and appropriate drug combinations is crucial. This study analyzes a dataset of lung disorder drugs, characterized by various topological indices. The structures of 16 drugs used to treat lung disorders are correlated with their physical properties using degree-based graph invariants. When considering specific attributes, the VIKOR (VlseKriterijumska Optimizacija I Kompromisno Resenje) method provides an optimal ranking for each drug. The QSPR results highlight the effectiveness of this approach in drug prioritization, offering valuable insights for clinical decision-making and drug development. This methodology can enhance the strategic selection of treatments for lung disorders, leading to improved patient care and better resource allocation.

## 1 Introduction

Human health is of utmost importance as it directly influences overall wellbeing and quality of life. Numerous diseases can significantly impact human health, and among these, lung disorders stand out as chronic respiratory conditions characterized by airway inflammation and bronchoconstriction. Over a quarter of a billion people worldwide suffer from lung disorders, making it the most common chronic illness in children. To address this condition, various drugs have been developed, including bronchodilators and anti-inflammatory agents, which help manage symptoms and reduce exacerbations. Recent advancements in medical research have utilized chemical graph theory to improve drug selection for lung disorder treatment, enabling a more precise and personalized approach to medication (Levy et al., 2023).

Chemical graph theory, at the intersection of chemistry and mathematics, employs graph theory principles to model molecules as graphs, with atoms as nodes and chemical bonds as edges. This approach enables the analysis of molecular structure, connectivity, and properties (Gutman et al., 2017). Recent applications include utilizing graph neural networks for molecular property prediction (Xiong et al., 2020) and investigating graph-theoretical descriptors for drug discovery and materials science (Lusci et al., 2013). A graph H=(V(H),E(H)) is an ordered pair with vertex set V(H) and edge set E(H) . The valency of vertices u and v is denoted as $d_{u}$ and $d_{v}$ respectively.

Topological indices, essential in chemical graph theory, are numerical descriptors that encapsulate the structural details of molecules using graph-based representations (Yao et al., 2020). These indices, calculated from molecular graphs where atoms serve as nodes and bonds as edges, are crucial for predicting diverse chemical and biological properties (Todeschini et al., 2016). Recent research underscores their application in drug design, materials science, and environmental chemistry (Gao et al., 2021), demonstrating their ongoing importance in elucidating structure-property relationships in complex molecular systems.

Degree based topological indices have potential application in understanding the molecular basis of any drugs. Some latest research works has explored the use of topological indices in the prediction of allergenicity and lung disorders risk related drugs (Nandy et al., 2020; Zheng et al., 2021). They aid in optimizing molecular structures to enhance pharmacological activity and bio-availability (Gasteiger and Marsili, 1980). On the whole, topological indices are used as versatile tools with diverse applications, ranging from chemistry and drug design to network analysis and environmental studies. Recent studies have applied Quantitative Structure-Property Relationship (QPSR) approaches to predict lung disorders triggering potential and the toxicity of chemicals linked to lung disorders development (Kumar et al., 2021; Mukherjee et al., 2021).

Multi-Criteria Decision Making (MCDM) methods are increasingly relevant in the context of QPSR analysis for drugs-related research. Recent studies have employed MCDM techniques to weigh and prioritize multiple criteria or properties when evaluating the potential asthma triggering or therapeutic capabilities of chemical compounds (Oliveira et al., 2020; Zheng et al., 2021).

In the realm of MCDM, VlseKriterijumska Optimizacija I Kompromisno Resenje (VIKOR) has gained recent prominence as a valuable tool for addressing complex decision-making challenges. The VIKOR method depends on a compromise programming technique-derived aggregating function that symbolizes“closeness to the ideal”. Its application extends to various domains, including healthcare and environmental management (Ishizaka et al., 2021).

The VIKOR method, a Multi-Criteria Decision Making (MCDM) technique, offers several strengths and weaknesses. One of its primary strengths is its ability to identify a compromise solution that is closest to the ideal, balancing various conflicting criteria effectively. This makes it particularly useful in scenarios where decision-makers have differing preferences and objectives (Kuchta et al., 2020). Additionally, VIKOR’s flexibility allows for the adjustment of parameters to reflect the decision-makers’ preferences, making it widely applicable across diverse fields such as engineering, management, and healthcare. The method’s relatively straightforward computational process also enhances its accessibility and ease of use (Erdebilli et al., 2023). However, VIKOR also has notable weaknesses. The method’s results are highly sensitive to the weights assigned to different criteria, which can introduce subjectivity and potential bias if the weights are not accurately determined. Furthermore, VIKOR requires precise and accurate data for its analysis, which may not always be available or easy to measure, limiting its applicability in situations with uncertain or vague data (Arif et al., 2020).

*Rest of the Paper:* The rest of the paper is organized as follows. Section 2. Describes all the existing works related to this research work. In section 3., findings of topological indices and application of VIKOR is explained in detail. Section 4. Wraps up the discussion by summarizing the results and synthesizing the key points.

## 2 Existing works

In this section, we delve into the historical context and recent advancements associated with QSPR analysis, particularly its integration with chemical graph theory and the implementation of MCDM methods in factual scenarios.

Novel topological properties of chemical structures have found applications in the treatment of COVID-19 (Mondal et al., 2022). In another study, various molecular drug structures, including hyaluronic acid, an anticancer drug, polyomino chains of n-cycles are subjected to analysis using MCDM techniques like Technique for Order of Preference by Similarity to Ideal Solution (TOPSIS) and Simple Additive Weighting (SAW) (Zheng et al., 2019). Further investigations involve the exploration of topological indices associated with hyaluronic acid (Ravi and Desikan, 2023). Graph operations, specifically the double and strong double graph, are employed to derive closed formulas for certain degree-based topological indices of silicon carbide (Sardar et al., 2023). The Nirmala leap index and modified Nirmala leap index of some chemical drugs are considered against COVID-19 (Kumar et al., 2023). Additionally, the face index formula is computed for specific molecular structures within the benzenoid series, such as Polycyclic Aromatic Hydrocarbons (PAHs), jagged-rectangle benzenoid systems, and concealed non-kekulean benzenoid systems (Joita et al., 2023).

An array of QSPR analyses have been conducted, each targeting specific areas of pharmaceutical research. In one instance, an association between electronvolts (eV) and degree-based topological descriptors is established to predict the molecular weight and topological polar surface area of phytochemicals (Lubbad, 2009). Additional investigations encompass degree-based and neighborhood degree sum-based topological indices for anti-HIV drugs using M-polynomial formulations (Huilgol et al., 2022), degree-based topological indices and regression models for nine anti-malaria drugs (Zhang et al., 2022), and the computation of reformulated leap Zagreb indices, leap eccentric connectivity indices, and reformulated Zagreb connectivity indices for antiviral drugs (Ravi et al., 2023). Moreover, the physicochemical and pharmacokinetic properties like Absorption, Distribution, Metabolism, Excretion, and Toxicity (ADMET) of anti-flaviviral drugs are forecasted through a QSPR model based on multiple Revan indices (Tamilarasi and Balamurugan, 2023; Tayebi et al., 2023). The application of M-polynomial methods in QSPR analysis is extended to study various degree-based topological indices for headache drugs such as naproxen, flurbiprofen, fenoprofen, ketoprofen, and ibuprofen (Sardar and Ali, 2022), and to investigate the relationships between topological indices and physicochemical properties of blood cancer treatment drugs (Parveen et al., 2023). Thirteen degree-based topological indices for anticancer drugs are also subject to QSPR analysis (Shanmukha et al., 2020). Additionally, QSPR domination indices are leveraged to create robust models for predicting the physicochemical properties of organic compounds while maintaining symmetry (Wazzan and Ahmed, 2023). Lastly, statistical analysis is harnessed to predict the properties of antiviral drugs without the need for experimental trials (Sivakumar and Rajkumar, 2023).

In various decision-making scenarios, distinct methods are employed to address complex multi-criteria challenges. One such method is the Criteria Weight Average VIKOR (CWA-VIKOR), designed to handle uncertain fuzzy situations by considering criteria weight averages and hesitant fuzzy preference data (Wan et al., 2020). Another approach, the extended Dual Probabilistic Linguistic-vise-VIKOR (DPL-VIKOR) method, is proposed for addressing multi-criteria group decision-making problems in risk assessment, particularly in Technological Innovation Project (TIP) (Ashraf et al., 2023). To aid in selecting health insurance plans, a hybrid fuzzy MCDM method is recommended (Erdebilli et al., 2023). Additionally, a solution involving the integration of VIKOR with the Simple Multi-Attribute Rating Technique Exploiting Ranks (SMARTER) method is presented for prioritizing goods sellers in an e-marketplace within a MCDM framework (Arif et al., 2020; Wan et al., 2023). In the context of evaluating security policies and content analysis of press agencies in Gulf countries, a Fuzzy VIKOR approach is introduced as a valuable MCDM technique. This method incorporates linguistic variables to mitigate uncertainties and subjectivity in expert decision-making processes (Talib, 2020).

(Hui et al., 2023) used VIKOR, TOPSIS and COPRAS for comparative ranking of the nano-tubes while establishing a solid connection between topological indices and physiochemical properties of nano-tubes. Multiple linear regresion has been done of carbon, naphthalene, boron nitride, V-phenylene, and titania nanotubes (Zaman et al., 2023). made a QSPR model to predict the properties flash point, molar volume, boiling point, molecular wight and complexity and demonstrated that QSPR play important role in drug discovery (Bulut et al., 2024). study aimed to evaluate the data according to five accepted criteria for the effects of twenty promising anticancer agents and rationalizes decision making in a fuzzy environment to avoid the high cost and time requirements of further pre-clinical and clinical studies. Also, the results of inhibition against both cancer cells and bacterial strains were confirmed by molecular docking calculation sand the results obtained in cancer studies were evaluated with a multi-criteria decision making methodology (MCDM) (Aydın et al., 2021). (Özcan et al., 2020) considered the problem of selecting the most promising anticancer agents, showing inhibition at low $IC_{50}$ concentration and low releasing lactate dehydrogenase percentage (cytotoxicity). All related abbreviations are shown in Table 1.

**TABLE 1:** Common Abbreviations.

Abbreviations	Definitions
QSPR	Quantitative Structure-Property Relationship
MCDM	Multi-Criteria Decision Making
VIKOR	VlseKriterijumska Optimizacija I Kompromisno Resenje
Epinephrine	(EP)
BP	Boiling Points
EV	Enthalpy of Vaporization
RA(H)	Randi $\overset{´}{c}$ Index
ABC(H)	Atom Bond Connectivity Index
$M_{1}\left(H\right)$	First Zagreb Index
$M_{2}\left(H\right)$	Second Zagreb Index
SCI(H)	Sum Connectivity Index
F(H)	Forgotten Index
GA(H)	Geometric Arithmetic Index
H(H)	Harmonic Index
HM(H)	Hyper Zagreb Index
$S_{j}$	Weighted normalized Manhattan distance
$R_{j}$	Weighted normalized Chebyshev distance
$Q_{j}$	VIKOR Index
SAW	Simple Additive Weighting

The authors have proposed a novel methodology to address these identified gaps, specifically applying QSPR analysis to investigate the characteristics of drugs for lung disorders. The primary contributions of this proposed work are summarized as follows:

• We have tabulated the chemical indices and their formulas in Table 2 and numerical computations of topological indices of drug structures Table 3.

• Subsequently, we have derived outcomes generated from regression analysis using Statistical Package for the Social Sciences (SPSS) within the framework of QSPR modeling.

• Two technique-based assessments VIKOR and SAW are conducted to rank the drugs, utilizing the comprehensive data presented in both charts and tables.

**TABLE 2:** Names of Chemical Indices employed in QSPR modeling.

Chemical indices	Formula
RA(H)	$\sum_{u,v\epsilon E\left(H\right)}\sqrt{\frac{1}{d_{u}d_{v}}}$
ABC(H)	$\sum_{u,v\epsilon E\left(H\right)}\sqrt{\frac{d_{u}+d_{v}-2}{d_{u}d_{v}}}$
$M_{1}\left(H\right)$	$\sum_{u,v\epsilon E\left(H\right)}d_{u}+d_{v}$
$M_{2}\left(H\right)$	$\sum_{u,v\epsilon E\left(H\right)}d_{u}d_{v}$
SCI(H)	$\sum_{u,v\epsilon E\left(H\right)}\sqrt{\frac{1}{d_{u}+d_{v}}}$
F(H)	$\sum_{u,v\epsilon E\left(H\right)}\left({\left(d_{u}\right)}^{2}+{\left(d_{v}\right)}^{2}\right)$
GA(H)	$\sum_{u,v\epsilon E\left(H\right)}\frac{2\sqrt{d_{u}d_{v}}}{d_{u}+d_{v}}$
H(H)	$\sum_{u,v\epsilon E\left(H\right)}\frac{2}{d_{u}+d_{v}}$
HM(H)	$\sum_{u,v\epsilon E\left(H\right)}{\left(d_{u}+d_{v}\right)}^{2}$

**TABLE 3:** Numerical results of topological indices.

Drug Name	ABC(H)	RA(H)	M_1_(H)	M_2_(H)	HM(H)	H(G)(H)	S(G)(H)	F(G)(H)	GA(H)
*Prednisone*	21.41348	12.52244	154	191	860	11.49048	12.82616	478	26.90294
*Methyle Prednisolone*	22.18954	12.93312	160	199	896	11.82381	13.2344	498	27.76896
*Prednisolone*	23.2506	13.27244	178	239	1052	12.24048	13.88682	574	29.90294
*Epinephrine*	9.439677	6.147066	60	67	286	5.833333	6.129915	152	12.43986
*Salbutamole*	12.63547	7.831517	82	90	406	7.266667	7.827018	226	15.89644
*Levosalbutamole*	11.2617	7.089935	71	75	345	6.533333	6.971556	195	13.91665
*Fluticasone*	24.68887	14.23589	186	246	1076	13.16429	14.85257	584	31.87819
*Salmeterol*	22.05821	14.70271	136	148	610	14.43333	14.91877	314	30.49302
*Flunisolide*	23.42047	14.21554	164	202	876	13.11905	14.39246	472	30.02918
*Ciclesonide*	33.0791	19.26968	237	293	1307	17.84048	19.93245	721	42.06883
*Mometasone*	28.53709	16.54184	217	291	1251	15.65952	17.56746	669	38.16604
*Vilanterol*	19.37961	13.31373	114	118	490	13	13.22167	254	26.43986
*Formoterol*	18.63207	12.02841	120	135	568	11.6	12.23219	298	25.20084
*Beclometasone*	29.7222	17.54151	220	288	1258	16.29286	18.13098	682	38.53254
*Montelukast*	15.50434	10.01164	95	99	441	9.5	9.960813	243	19.97726
*Zileutone*	12.29661	7.592224	82	96	406	7.233333	7.816275	214	16.34288

## 3 Materials and methods

**FIGURE 1:**
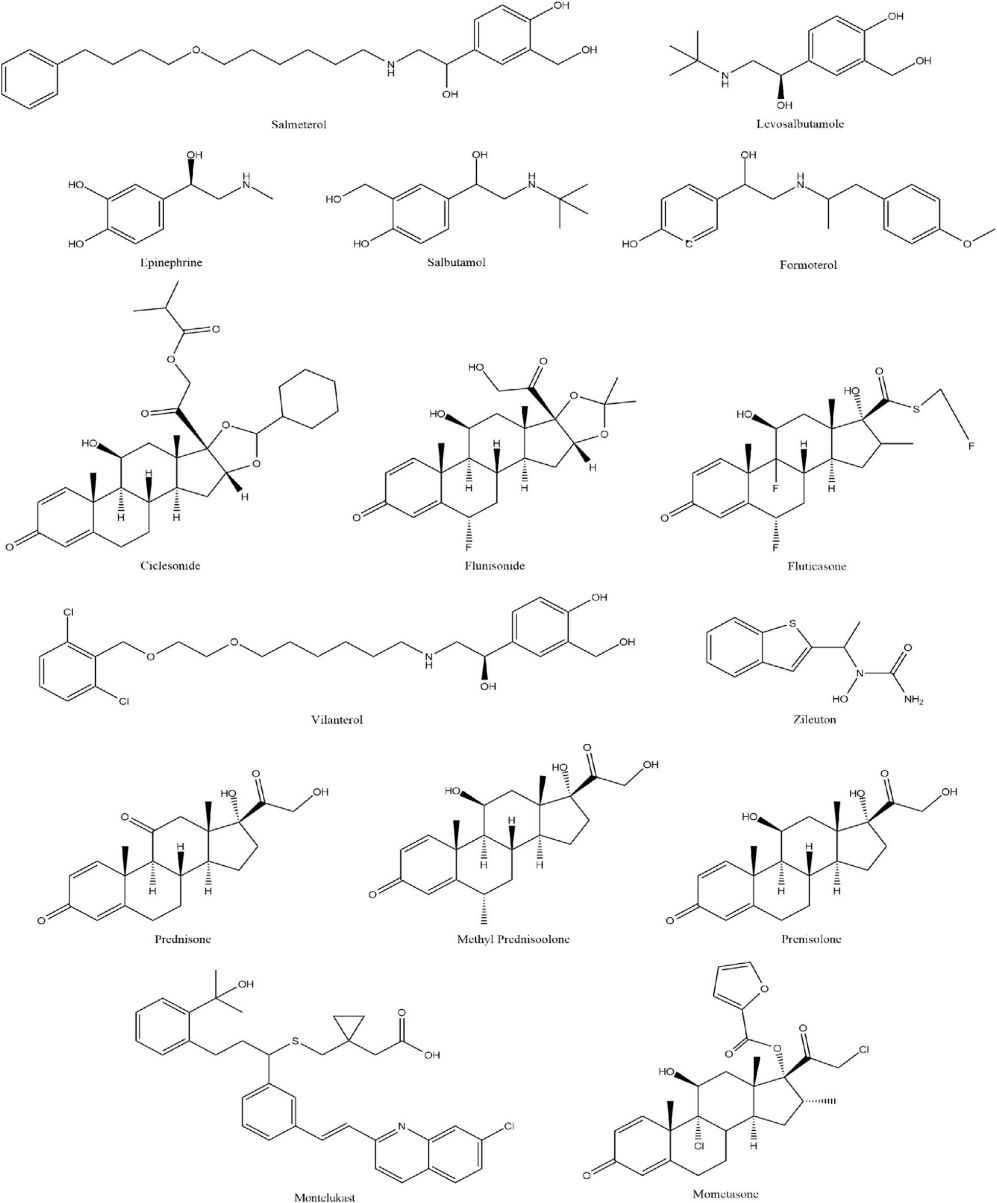
Drugs used for the treatment of Lung Disorders.

Lung diseases are characterized by inflammation and muscle constriction around the airways, manifests symptoms such as coughing, wheezing, shortness of breath, and chest tightness. Inhaled corticosteroids, Long-acting beta2 agonists, Leukotriene receptor antagonists and Leukotriene inhibitors the most effective long-term medications for asthma. These medications include Ciclesonide, Epinephrine (adrenaline), Flunisolide, Fluticasone, Formoterol, Levosalbutamole, Methyl Prednisolone, Mometasone, Montelukast, Prednisolone, Beclometasone, Prednisone, Salbutamole, Salmeterol, Vilanterol, and Zileutone (Falk et al., 2016). This study evaluates 16 drugs for lung disorders as shown in Figure 1.

The efficacy of asthma drugs is closely tied to their chemical compositions, which determine their mechanisms of action. Inhaled corticosteroids (ICS) like ciclesonide, flunisolide, fluticasone, beclometasone, and mometasone use fluorinated steroid structures to provide potent local anti-inflammatory effects with minimal systemic absorption, effectively controlling chronic symptoms (Nandy et al., 2020). Epinephrine, a non-selective catecholamine, offers rapid bronchodilation during acute attacks by activating both alpha and beta adrenergic receptors. Selective beta-2 adrenergic agonists, including short-acting salbutamol (albuterol) and levalbuterol, as well as long-acting formoterol, salmeterol, and vilanterol, relax bronchial smooth muscle to induce bronchodilation, with efficacy influenced by their chemical structures. Systemic corticosteroids like methylprednisolone, prednisolone, and prednisone provide potent anti-inflammatory effects crucial for severe exacerbations (Kumar et al., 2021). Montelukast and zileuton target leukotriene pathways to reduce bronchoconstriction and inflammation, effectively managing chronic asthma through their specific molecular configurations. Each drug’s unique composition underpins its efficacy, allowing for tailored asthma management that balances therapeutic benefits with side effects (Mukherjee et al., 2021).

Utilizing a MCDM approach for ranking based on QSPR analysis, the research emphasizes physical and structural properties, with a focus on boiling points (BPs) and enthalpy of vaporization (EV). Ratio weighting method (Odu, 2019) is used for weight allocation for the application of VIKOR that results in an optimal ranking for each lung disorders medication. Chemical indices used in QSPR modeling are detailed in Table 2 along with their formulas.

We have calculated the topological indices of a drug structure epinephrine (EP) having the edge partition.

$|E_{1,2}|=1,|E_{1,2}|=1,|E_{1,3}|=3,|E_{2,2}|=2,|E_{2,3}|=5,|E_{3,3}|=2$

While using the formulas mentioned in Table 2.

$RA\left(EP\right)=1\sqrt{\frac{1}{2}}+3\sqrt{\frac{1}{3}}+2\sqrt{\frac{1}{4}}+5\sqrt{\frac{1}{6}}+2\sqrt{\frac{1}{9}}=6.14706$

$ABC\left(EP\right)=1\sqrt{\frac{1}{2}}+3\sqrt{\frac{2}{3}}+2\sqrt{\frac{2}{4}}+5\sqrt{\frac{3}{6}}+2\sqrt{\frac{4}{9}}=9.43967$

$M_{1}\left(EP\right)=1\left(3\right)+3\left(4\right)+2\left(4\right)+5\left(5\right)+2\left(6\right)=60$

$M_{2}\left(EP\right)=1\left(2\right)+3\left(3\right)+2\left(4\right)+5\left(6\right)+2\left(9\right)=67$

$SCI\left(EP\right)=1\sqrt{\frac{1}{3}}+3\sqrt{\frac{1}{4}}+2\sqrt{\frac{1}{4}}+5\sqrt{\frac{1}{5}}+2\sqrt{\frac{1}{6}}=6.14706$

F(EP)=1(5)+3(10)+2(8)+5(13)+2(18)=152

$GA\left(EP\right)=1\left(2\frac{\sqrt{2}}{3}\right)+3\left(2\frac{\sqrt{3}}{4}\right)+2\left(2\frac{\sqrt{4}}{4}\right)+5\left(2\frac{\sqrt{6}}{5}\right)+2\left(2\frac{\sqrt{9}}{6}\right)=12.43986$

$H\left(EP\right)=1\left(\frac{2}{3}\right)+3\left(\frac{2}{4}\right)+2\left(\frac{2}{4}\right)+5\left(\frac{2}{5}\right)+2\left(\frac{2}{6}\right)=5.83333$

HM(EP)=1(9)+3(16)+2(16)+5(25)+2(36)=286

All the remaining topological indices of each drug are calculated in similar way and numerical results of topological indices are mentioned in Table 3.

### 3.1 Correlation coefficient between physical properties and indices

We specifically examine the standard error (SE) and correlation coefficients (r) values considering properties with chemical indices, as detailed in Table 4, 5.

**TABLE 4:** Standard error and correlation between indices and BP.

Chemical indices	Standard Error (SE)	Correlation (r)
RA(H)	76.02092616	0.615
ABC(H)	70.83996637	0.678
$M_{1}\left(H\right)$	82.10335013	0.524
$M_{2}\left(H\right)$	86.03267444	0.451
SCI(H)	86.40526283	0.443
F(H)	69.49911131	0.693
GA(H)	72.63612711	0.657
H(H)	86.75378512	0.436
HM(H)	75.03459712	0.628

**TABLE 5:** Standard error and correlation between indices and EV.

Chemical indices	Standard Error (SE)	Corelation (r)
RA(H)	9.64037899	0.754
ABC(H)	9.06843885	0.786
$M_{1}\left(H\right)$	10.5989982	0.692
$M_{2}\left(H\right)$	11.34996569	0.634
SCI(H)	11.40039383	0.63
F(H)	9.0606885	0.787
GA(H)	9.30315409	0.774
H(H)	11.45839242	0.625
HM(H)	9.62001605	0.755

The proximity of the correlation coefficient (r) to 1, along with insignificant standard error, signifies the strong predictive potential of the respective chemical indices for the properties of interest. This concept underscores the importance of both r and SE in assessing the capability of selected chemical indices to forecast the physiochemical properties of drugs.

### 3.2 Weight allocation

This research focuses on categorizing drugs based on their physio-chemical properties, particularly by extracting QSPR data for EV and BPs. The study emphasizes that drug solubility plays a crucial role in their effectiveness, as more soluble drugs tend to be more potent. It also highlights the impact of endothermic substances on the dissolution process by absorbing heat during dis-solution. Lower melting and BPs are associated with greater positive effects for drugs, while high BPs can lead to reduced drug impact due to evaporation.

**FIGURE 2:**
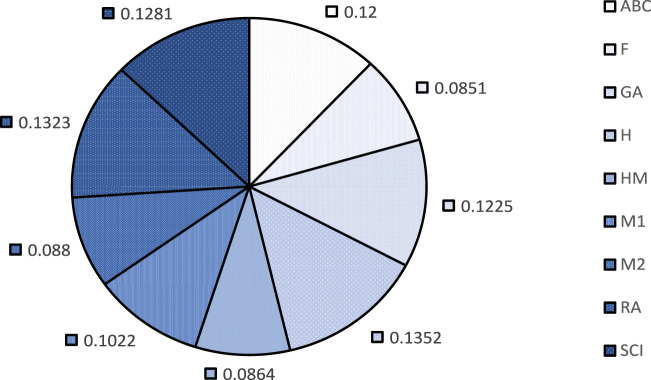
Allocated weights for BP.

**FIGURE 3:**
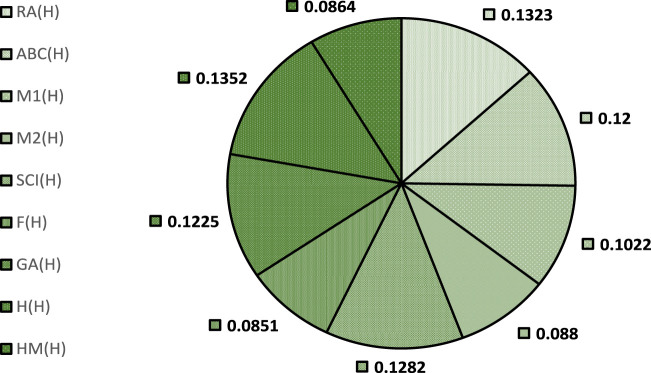
Allocated weights for EV.

To facilitate this ranking, we designate the correlation coefficient as the weighting criterion. We have used ratio weighting method (Odu, 2019) for weight allocation using the formula $w_{i}=\frac{r_{i}}{\sum r_{i}}$ for BP in Figure 2 and for EV in Figure 3. These weights are categorized into beneficial and non-beneficial criterion accordingly, $w_{i}>0.10$ are considered beneficial and $w_{i}\leq 0.10$ are non-beneficial (Li et al., 2022). This method is selected because it is a subjective weighting approach that relies on decision makers to rank the relevant criteria based on their importance.

### 3.3 VIKOR technique

To achieve the best possible outcome close to the optimal result, we employ the VIKOR technique. In this approach, drugs treated as alternatives, are evaluated based on predefined criteria derived from the QSPR analysis conducted in the case study. VIKOR is used to rank these drugs and determine the compromise solution that comes closest to the ideal outcome. The concept of compromise solutions in MCDM was initially introduced by Zeleny (Sivakumar and Rajkumar, 2023) and Yu (Wan et al., 2020) in 1973, with practical applications identified in 1998 (Ashraf et al., 2023). The offered technique involves a series of steps outlined in Algorithm 1.If it is compared with other existing MCDM methods such as TOPSIS, which requires same number of properties and drugs for ranking drugs, the computational complexity increases (Hui et al., 2023) See Tables 6, 7.

**TABLE 6:** VIKOR results of $S_{j},R_{j},Q_{j}$ and Ranks of drugs for BPs.

Lung disorders drugs	$S_{j}$	$R_{j}$	$Q_{j}$	BP rank
Prednisone	0.527392105	0.071511068	0.397714246	10
Methyleprednisolone	0.520804376	0.067757243	0.362124907	7
Prednisolone	0.54296303	0.068162602	0.404753247	11
Epinephrine	0.638243902	0.135219512	1	16
Salbutamole	0.601727818	0.119077899	0.832156082	13
Levosalbutamole	0.618575117	0.127336402	0.914710696	15
Fluticasone	0.509280745	0.072783795	0.372989036	8
Salmeterol	0.361888448	0.05594497	0.000239342	1
Flunisolide	0.464835707	0.060075513	0.212461108	3
Ciclesonide	0.361756098	0.102243902	0.292016399	4
Mometasone	0.450132572	0.090690919	0.378969321	9
Vilanterol	0.394635128	0.069542256	0.145219109	2
Formoterol	0.464735555	0.073336968	0.295922571	5
Beclometasone	0.423257793	0.092423867	0.341299062	6
Montelukast	0.519858807	0.093927028	0.52547288	12
Zileutone	0.605031291	0.119453293	0.840497758	14

**TABLE 7:** VIKOR results of $S_{j},R_{j},Q_{j}$ and Ranks of drugs for EV.

Lung disorders drugs	$S_{j}$	$R_{j}$	$Q_{j}$	EV rank
Prednisone	0.528974263	0.064658425	0.426815195	6
Methyleprednisolone	0.524923022	0.061264316	0.393204055	5
Prednisolone	0.554421627	0.07495934	0.556502937	11
Epinephrine	0.599036818	0.122261923	1	16
Salbutamole	0.571980933	0.107667101	0.835191937	13
Levosalbutamole	0.584089984	0.115134223	0.915864042	15
Fluticasone	0.524607346	0.078010011	0.516302268	9
Salmeterol	0.361462583	0.054609429	0	1
Flunisolide	0.472049044	0.063165896	0.295979225	3
Ciclesonide	0.400963182	0.107503495	0.474057902	7
Mometasone	0.479851685	0.097621467	0.567051853	12
Vilanterol	0.384906253	0.067882115	0.147434254	2
Formoterol	0.454388706	0.071586238	0.321043495	4
Beclometasone	0.455272446	0.097178301	0.512046946	8
Montelukast	0.498069192	0.08745275	0.530238473	10
Zileutone	0.575119521	0.108659305	0.849130515	14

Algorithm 1 VIKOR Technique.

1: Determination of ideal best $f_{i}^{+}$ and ideal worst $f_{i}^{-}$ values where
          
{i=Pi,i=1,…,n}

 for all criterion functions which we considered as predicted properties.
 
$f_{i}^{+}=\left\{max\left\{f_{ij},J=1,\ldots ,m\right\},min\left\{f_{ij},J=1,\ldots ,m\right\}:\right.$ if the $i^{th}$ function is beneficial}.
 
$f_{i}^{-}=min\left\{f_{ij},J=1,\ldots ,m\right\},max\left\{f_{ij},J=1,\ldots ,m\right\}$ : if the $i^{th}$ function is unbeneficial}
2: Determination of the values of $S_{j}$ (weighted normalized Manhattan distance) and Rj (weighted normalized Chebyshev distance) where j=1,…,m . We have the following inequalities.
          
$S_{j}=\sum_{j=1}^{m}\left[w_{i}\times \frac{\left(f_{i}^{+}-f_{ij}\right)}{\left(f_{i}^{+}-f_{i}^{-}\right)}\right]$ ,
          
$R_{j}=max\left[w_{i}\times \frac{\left(f_{i}^{+}-f_{ij}\right)}{\left(f_{i}^{+}-f_{i}^{-}\right)}\right]$

3: Determination of values $Q_{j},\;j=1,\ldots ,J$ , through the following equality
          
$Q_{j}=\left[v\times \frac{\left(S_{j}-S^{+}\right)}{\left(S^{-}-S^{+}\right)}\right]+\left[\left(1-v\right)\times \frac{\left(R_{j}-R^{+}\right)}{\left(R^{-}-R^{+}\right)}\right]$

 whereas (1−v) is the weight of the individual regret. This strategy could be compromised by v=0.5

4: Rank the alternatives, sorting by the values S,R and Q from the minimum value

### 3.4 Simple Additive Weighting (SAW)

SAW is a widely used multicriteria decision-making method that involves assigning weights to criteria and then summing up the weighted scores of alternatives to rank them (Zheng et al., 2019). Here’s how SAW works:

1. Normalization: The first step is to normalize the decision matrix, which contains the performance of each alternative across all criteria. Normalization ensures that all criteria are on the same scale and allows for fair comparison.

2. *Weighting:* Next, weights are assigned to each criterion to reflect their relative importance in the decision-making process. These weights can be determined based on the preferences of decision-makers, stakeholder consultations, or other methods such as the entropy method.

$$\text{Weighted Score}\left(S_{ij}\right)=x_{ij}\times w_{j}$$

3. *Scoring:* Each alternative is scored on each criterion by multiplying its performance value by the corresponding weight. This results in a weighted score for each alternative on each criterion.

$$\text{Total Score}\left(TS_{i}\right)=\overset{n}{\underset{j=1}{\sum }}S_{ij}$$

4. *Aggregation:* The weighted scores for each alternative are then aggregated by summing them up across all criteria. This results in a total score for each alternative.

5. *Ranking:* Finally, the alternatives are ranked based on their total scores, with higher scores indicating better performance. See Table 8.

**TABLE 8:** SAW ranks.

Drug Name	O_i_^*^	Rank
*Prednisone*	0.88645496	16
*MethylPrednisolone*	0.38838398	5
*Prednisolone*	0.7819411	15
*Epinephrine*	0.36717549	1
*Salbutamol*	0.42259203	7
*Levosalbutamole*	0.42019067	6
*Fluticasone*	0.68518714	14
*Salmeterol*	0.38523964	4
*Flunisolide*	0.42878317	8
*Ciclesonide*	0.4964753	9
*Mometasone*	0.52065274	10
*Vilanterol*	0.56131761	11
*Formoterol*	0.62506854	12
*Beclometasone*	0.37310154	2
*Montelukast*	0.3750034	3
*Zileutone*	0.67003225	13

SAW is straightforward and easy to implement, making it a popular choice for decision-making in various fields such as business, finance, and project management. However, it does not account for interactions between criteria and may not always produce the most optimal solution.

## 4 Conclusions and future directions

In this paper, 16 lung disorders drugs used in chemotherapy combinations were analyzed using VIKOR, a MCDM technique. The VIKOR analysis heavily relies on evaluations and considers two properties: BP and EV, in a QSPR modeling context. This approach is employed in drug research, focusing on two properties, i.e., BP and EV, significantly affecting drug absorption. In our QSPR analysis, we used correlation coefficients to relate individual properties to targeted degree-based chemical indices, along with error values from the evaluations. Degree-based chemical indices were chosen for their strong predictive capability.

**FIGURE 4:**
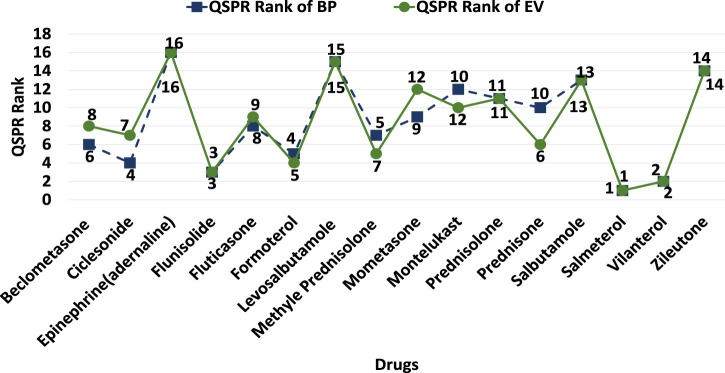
Ranks of lung disorders treatment drugs obtained by employing VIKOR.

We conducted evaluations using a decision-making technique to address this new research focus. The resulting rankings for 16 specific drugs based on their BP and EV are presented in Figure 4. These rankings provide valuable insights for scientists and chemists seeking to create effective drug combinations. Notably, eight out of the 16 lung disorders drugs achieved the same rankings in both the VIKOR process, considering BP and EV as provided in Table 7, 8, respectively. These drugs, along with their rankings, are as follows: Salmeterol (Rank 1), Vilantrol (Rank 2), Flunisolide (Rank 3), Prednisolone (Rank 11), Salbutamole (Rank 13), andZileutone (Rank 14), Levosalbutamole (Rank 15), and Epinephrine (Adrenaline) (Rank 16). This suggests that considering both BP and EV in the evaluation leads to equivalent rankings for these eight drugs.

**FIGURE 5:**
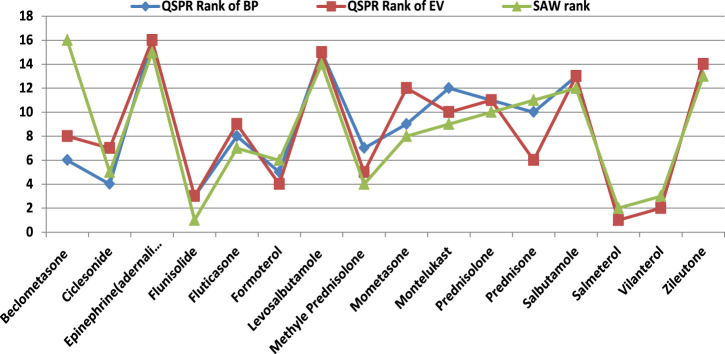
VIKOR and SAW ranks.

Furthermore, SAW is employed and ranks obtained from this MCDM method are compared with VIKOR ranks in Figure 5.

This research aims to draw conclusions from the QSPR analysis regarding two physio-chemical properties, BP, and EV, with implications for drug development and solubility estimation in medicinal and environmental chemistry. It demonstrates how QSPR evaluations impact the ranking of multiple structures under various criteria. It’s essential to keep in account that the emphasis is not solely on chemical indices but also on how individual chemical indices contribute to achieving the best outcomes, offering insights for biologists and scientists to explore potential drug combinations. The proposed strategy of relating indices and properties can be employed for drug discovery and predicting properties.

## Data Availability

The original contributions presented in the study are included in the article/supplementary material, further inquiries can be directed to the corresponding author.
